# The Distinction between Instinct and Reason; the Introductory Discourse to a Series of Lectures on the Properties and Functions of Animal Life, Delivered before the Philosophical and Literary Society at Portsmouth

**Published:** 1844-04

**Authors:** 


Art. III.—*The Distinction between Instinct and Reason. The Introductory Discourse to a Series of Lectures on the Properties and Functions of Animal Life, delivered before the Philosophical and Literary Society at Portsmouth.* By J. Strang, m.d., Vice-president of the Society.—*London* 1843. 8vo, pp. 44.

A very sensible and well-argued popular disquisition upon a knotty question, taking a medium view between the extreme opinions which have been upheld upon the subject, that corresponds closely with the one which we advocated in our Eleventh Volume.

